# A Meta-Analysis on Predictors of Mortality Among Patients Hospitalized for Acute Exacerbation of Asthma

**DOI:** 10.7759/cureus.35225

**Published:** 2023-02-20

**Authors:** Abdulmaleek Idanesimhe Sado, Muhammad S Afzal, Lavanya Kannekanti, Hrushikesh Reddy Pamreddy, Jorge Pimentel Campillo, Vaishnavi Kandukuri, Gnana Deepthi Medarametla, Sujith K Palleti

**Affiliations:** 1 Department of Medicine, Penwortham St Mary's Medical Group, Preston, GBR; 2 Department of Medicine, Carle Foundation Hospital, Urbana, USA; 3 Department of Medicine, Osmania Medical College, Hyderabad, IND; 4 Department of Internal Medicine, Osmania Medical College, Hyderabad, IND; 5 Department of Internal Medicine, CEDIMAT, Santo Domingo, DOM; 6 Department of Internal Medicine, Gandhi Medical College and Hospital, Hyderabad, IND; 7 Department of Internal Medicine, NRI Medical College and Hospital, Guntur, IND; 8 Department of Nephrology, Edward Hines Jr. Veterans Administration Hospital, Hines, USA; 9 Department of Nephrology, Loyola University Medical Center, Maywood, USA

**Keywords:** meta-analysis, exacerbation, mortality, predictors, asthma

## Abstract

The aim of this meta-analysis is to systematically review published studies and identify clinically important factors predicting mortality among patients hospitalized for acute exacerbation of asthma. This study was a meta-analysis conducted in accordance with the MOOSE (Meta-analysis of Observational Studies in Epidemiology) guidelines. A systematic search was carried out on online databases such as PubMed and EMBASE to identify articles on predictors of mortality among patients hospitalized for acute exacerbation of asthma. The search used keywords such as "asthma," "exacerbation," "mortality," and "factors." A total of six articles met the inclusion criteria and were included in the present meta-analysis. The incidence of short-term mortality among patients hospitalized for acute exacerbation of asthma was 6% (95% CI= 3-9%, I-square=99%) with a range of 0.79% to 18% across the studies. The factors significantly associated with short-term mortality in patients hospitalized for acute exacerbation of asthma including diabetes mellitus (RR=2.02, 95% CI: 1.63-2.52, p-value=0.001), pneumonia (RR=3.71, 95% CI: 3.02-4.56, p-value=0.001), and mechanical ventilation (RR: 29.98, 95% CI: 15.46-58.15, p-value=0.001). The present study found that diabetes mellitus, pneumonia, and the use of mechanical ventilation are independently associated with mortality among patients hospitalized for acute exacerbation of asthma. Healthcare professionals need to understand the comorbidities and risk factors associated with mortality in patients hospitalized for acute exacerbation of asthma in order to identify patients who are at increased risk and provide prompt treatment.

## Introduction and background

Asthma is a highly prevalent and chronic respiratory illness affecting 300 to 400 million individuals worldwide [1]. It is caused by chronic inflammation of the respiratory tract and is characterized by episodes of coughing, shortness of breath, wheezing, and chest tightness [2]. Each year in the United States, more than 10 million patients have an acute worsening of respiratory symptoms, known as asthma exacerbation, after exposure to environmental irritants, allergens, or respiratory viral infections [3]. While the majority of asthma exacerbations are treated in an outpatient setting, more severe cases may require hospitalization, putting a significant burden on the healthcare system [4]. For patients experiencing asthma exacerbation upon arrival at the emergency department or upon hospital admission, it is important for physicians to have knowledge of risk factors related to mortality in order to provide appropriate treatment efforts.

Health statistics show a decrease in the number of deaths due to asthma globally in recent decades [5]. This reduction in asthma mortality can be attributed to the widespread use of inhaled corticosteroids (ICSs) as a treatment option over the past two to three decades [6]. However, the death rate among asthmatics remains high, potentially due to a failure to identify fatal risk factors in these patients [7].

Despite the recognition of the financial and health burden of severe asthma exacerbations, few studies have been conducted to determine the predictors of mortality among patients hospitalized for asthma exacerbations. Understanding disease prognosis and risk factors that predict poor outcomes is vital for physicians to be able to advise patients on the expected course of the disease and the likelihood of complications. This information is also important for guiding management decisions such as the intensity of monitoring, site of care, decisions to withdraw or escalate treatment, and follow-up timing after discharge. Identifying mortality predictors in asthma exacerbations will also be useful for improving patient care and directing resources for asthma, an increasingly common illness. Therefore, we are conducting a study to systematically review published studies and identify clinically significant factors predicting mortality among patients hospitalized for acute exacerbation of asthma.

## Review

Methods

This study was a meta-analysis conducted in accordance with the MOOSE (Meta-analysis of Observational Studies in Epidemiology) guidelines.

Search Criteria

A systematic search was carried out on online databases such as PubMed and EMBASE to identify articles on predictors of mortality among patients hospitalized for acute exacerbation of asthma. The search used keywords such as "asthma," "exacerbation," "mortality," and "factors." The search included articles published between 2000 and January 2023 and did not have any language criteria. The reference lists of included articles were also manually searched to identify any additional studies related to the topic of interest.

Study Selection

Studies were eligible for inclusion if they met the following criteria: (a) original studies and (b) inclusion of adults hospitalized with asthma exacerbation. Studies were excluded if they combined asthma exacerbations with other acute diagnoses, such as chronic obstructive pulmonary disease (COPD) exacerbation, or if they included alternative reasons for admission in a study subject with pre-existing asthma. Additionally, case reports, case series, review articles, and editorials were excluded.

The incidence of short-term mortality among patients hospitalized for acute exacerbation of asthma was assessed. Short-term mortality was defined as mortality occurring within 90 days of first presentation to the hospital with exacerbation, and it included any studies that considered in-hospital mortality as an endpoint.

Data Analysis

Data analysis was performed using Review Manager 4.5.1 (Cochrane Collaboration, Oxford, United Kingdom). To determine the association between categorical predictors and mortality, risk ratios (RR) were computed using a Mantel-Haenszel random effects model, comparing the prevalence of each predictor in survivors versus non-survivors. A cut-off of the p-value was set at 0.05. Heterogeneity among the study results was assessed using I-square statistics and the Cochran-Q test. A p-value less than 0.1 was considered to represent significant heterogeneity. For I-square, <25% showed low heterogeneity, 25% to 50% showed moderate heterogeneity, and >50% showed high heterogeneity.

Results

**Figure 1:**
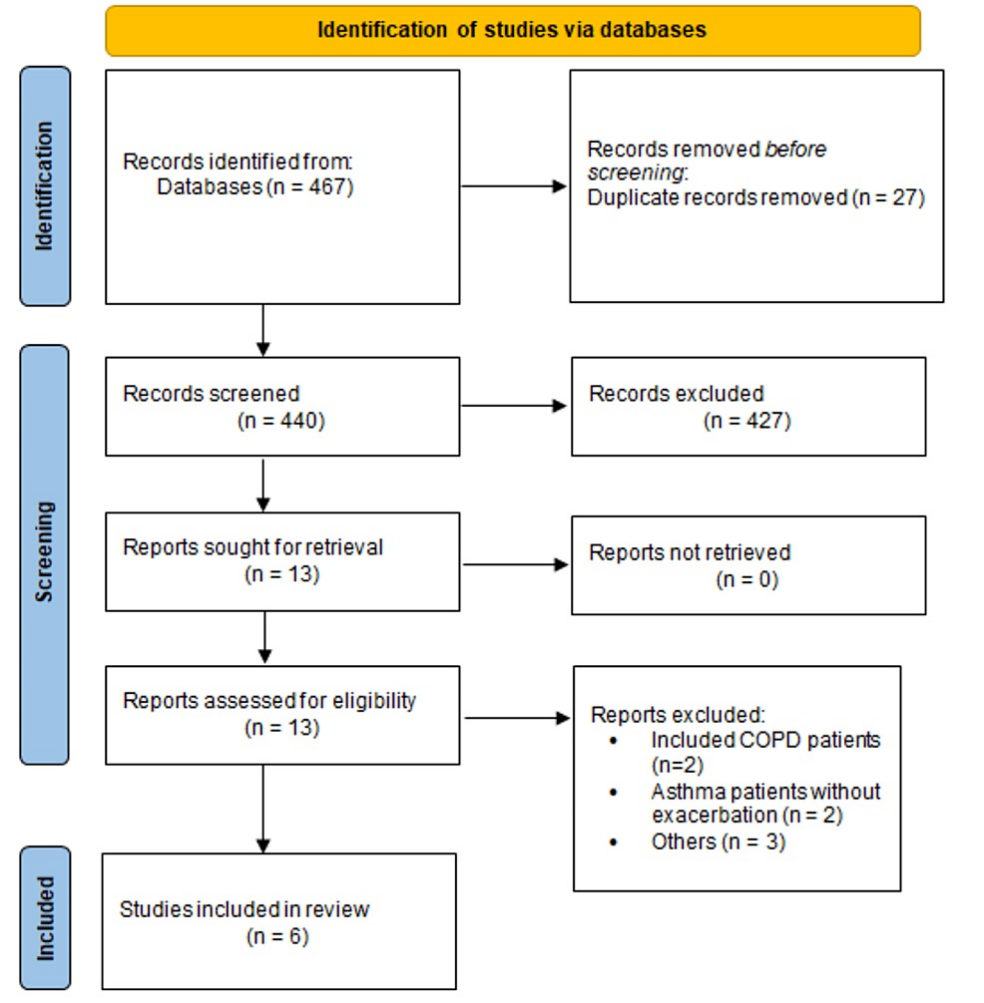
Flowchart of selection of studies COPD: chronic obstructive pulmonary disease

A total of 467 articles were retrieved through online database searches. After removing duplicates, 440 articles remained eligible for the title and abstract screening. Full texts of 13 articles were obtained and assessed for inclusion and exclusion criteria, and six articles met the criteria and were included in the present meta-analysis. Figure 1 presents the Preferred Reporting Items for Systematic Reviews and Meta-Analyses (PRISMA) flowchart of the study selection process. The characteristics of the included studies are summarized in Table 1.

**Table 1:** Characteristics of included studies

Author	Year	Study Design	Region	Sample Size
Chang et al. [8]	2020	Nested Case Control	Taiwan	124
Ekstrom et al. [9]	2020	Retrospective Cohort	Sweden	15697
Ibrahim et al. [10]	2023	Retrospective Cohort	Nigeria	124
Krishnan et al. [11]	2006	Retrospective Cohort	United States	65381
Para et al. [12]	2022	Retrospective Cohort	Italy	20056
Stefan et al. [13]	2015	Retrospective Cohort	United States	13930

Short-term Mortality

The results of five studies that assessed short-term mortality as an endpoint were pooled. The incidence of short-term mortality among patients hospitalized for acute exacerbation of asthma was 6% (95% CI=3-9%, I-square=99%) with a range of 0.79% to 18% across the studies. The factors associated with death are presented in Table 2.

**Table 2:** Predictors of mortality among patients hospitalized for acute exacerbation of asthma * significant at p-value< 0.05 RR: risk ratio; CI: 95% confidence interval

Predictors	Number of studies	RR (95% CI)	I-square
Diabetes Mellitus	2	2.02 (1.63-2.52)*	76%
				Pneumonia	2	3.71 (3.02-4.56)*	92%
Gender (Male)	2	0.99 (0.92-1.06)	0%				
				Need for Mechanical Ventilation	2	29.98 (15.46-58.15)*	84%

Diabetes

The impact of diabetes on mortality in patients hospitalized for acute exacerbation of asthma was assessed in two studies. The pooled prevalence of diabetes was 16.19% and the risk of death was significantly higher in diabetic patients (34.19%) compared to nondiabetic patients (16.23%) (RR=2.02, 95% CI: 1.63-2.52, p-value=0.001, I-square=76%).

Pneumonia

The impact of pneumonia on mortality in patients hospitalized for acute exacerbation of asthma was reported by two of the six included studies. The incidence of mortality was higher in patients with pneumonia (10.55%) compared to those without pneumonia (1.19%) and this difference was statistically significant (RR=3.71, 95% CI: 3.02-4.56, p-value=0.001, I-square: 92%).

Gender

There was no significant difference between males and females in terms of the risk of mortality (RR=0.99, 95% CI: 0.92-1.06, p-value=0.76, I-square: 0%).

Need of Mechanical Ventilation at the Time of Admission

The risk of mortality was significantly higher in patients receiving mechanical ventilation (invasive and non-invasive) compared to patients without a need for mechanical ventilation (RR: 29.98, 95% CI: 15.46-58.15, p-value: 0.001, I-square: 84%).

Discussion

This is the first study to review the literature on predictors of mortality in patients hospitalized for acute exacerbation of asthma. The overall incidence of short-term mortality among such patients was found to be 6%. A previous study by Singanayagam et al. reported an overall short-term cumulative incidence of mortality of 3.6% in patients admitted to the hospital due to COPD exacerbation [14].

This study found that individuals with diabetes mellitus have a 2.02 times higher risk of dying from asthma compared to those without diabetes, which is in line with previous studies that have linked diabetes to decreased lung capacity and changes in respiratory function. This finding is significant for healthcare providers as it emphasizes the importance of early screening for asthma in people with type 2 diabetes mellitus, and the need for prompt treatment when necessary, taking into account any modifiable risk factors [15,16].

Factors associated with mortality include a requirement for mechanical ventilation and concurrent pneumonia. Guite et al. conducted a study to investigate the risk factors for asthma-related deaths occurring within three years of discharge from hospital admission for asthma [17]. According to this study, the independent factors that increase the risk of death due to asthma include a previous history of severe asthma, chest pain, abnormal blood chemistry or hematology at the time of admission, being prescribed the drug ipratropium bromide, and not being prescribed ICSs at the time of discharge [17]. Watson et al. conducted a study using the United Kingdom database and reported that comorbidities of diabetes, cardiovascular diseases, and respiratory infection are common in older patients with asthma and may contribute to mortality after asthma-relation hospitalization [18]. The current meta-analysis identified that comorbidity conditions like pneumonia and diabetes were associated with an increased risk of mortality in patients hospitalized for asthma exacerbation. Thus, understanding comorbidities and risk factors is significant for healthcare professionals so they can identify patients who are at increased risk of mortality in the emergency department.

Menzies-Gow et al. proposed six key principles for addressing the under-treated problem of severe asthma: prompt diagnosis, proper referral, support for patients through education and personalized information about asthma, the treatment that does not rely solely on oral corticosteroids, and access to high-quality consistent care [19]. These strategies, informed by the latest understanding of asthma, should serve as a benchmark for evaluating current medical services. The results of this meta-analysis will help researchers to identify the literature gap and also emphasize the need for continued research in this area in order to better understand the factors that contribute to mortality in patients with acute exacerbations and to develop effective interventions for preventing and managing these events. In addition, the results will also help healthcare professionals to identify patients who are at greater risk of death and those who need specialized care. The results of this meta-analysis serve as a call to action for healthcare providers and policymakers to prioritize the prevention and management of acute exacerbations in order to improve patient outcomes and reduce the mortality rate.

Study limitations

The present meta-analysis has certain limitations, and the findings need to be interpreted with caution. Many of the studies only provided information on the frequency of certain risk factors and did not take into consideration the effect of the severity of the disease or death. Future studies should include adjustments based on factors such as age and gender when examining the relationship between comorbidities and death or severe disease. Some factors, such as tobacco smoking, were not assessed in the current meta-analysis due to a lack of relevant data. There are certain factors that were not assessed in the current meta-analysis because of a lack of relevant data. For instance, tobacco smoking is clinically important and significantly associated with mortality in patients hospitalized for acute exacerbation of asthma [10]. However, only one study assessed the relationship of this factor with mortality in this population, so we were not able to get a pooled estimate of this significant risk factor. Similarly, there are other risk factors as well that were only assessed by one study including the number of comorbidities, Charlson comorbidity score, duration of asthma, and medications used for the management of asthma at home. Studies are required in the future including larger sample size and assessing all clinically significant predictors of mortality in patients hospitalized for acute exacerbation of asthma.

## Conclusions

The incidence of short-term mortality among patients hospitalized for acute exacerbation of asthma was 6%. The present study found that diabetes mellitus, pneumonia, and the use of mechanical ventilation are independently associated with mortality among patients hospitalized for acute exacerbation of asthma. Healthcare professionals need to understand the comorbidities and risk factors associated with mortality in patients hospitalized for acute exacerbation of asthma in order to identify patients who are at increased risk and provide prompt treatment. Further studies are needed to improve the accuracy of these findings including a larger sample size to understand predictors of mortality in this population more clearly.
